# ERK mediated upregulation of death receptor 5 overcomes the lack of p53 functionality in the diaminothiazole DAT1 induced apoptosis in colon cancer models: efficiency of DAT1 in Ras-Raf mutated cells

**DOI:** 10.1186/s12943-016-0505-7

**Published:** 2016-03-08

**Authors:** Reshma Thamkachy, Rohith Kumar, K. N. Rajasekharan, Suparna Sengupta

**Affiliations:** Cancer Research Program - 3, Rajiv Gandhi Centre for Biotechnology, Trivandrum, 695014 India; Department of Chemistry, University of Kerala, Trivandrum, India

**Keywords:** *Diaminothiazoles*, *p53*, *Death Receptor 5*, *Ras/Raf/ERK*

## Abstract

**Background:**

p53 is a tumour suppressor protein that plays a key role in many steps of apoptosis, and malfunctioning of this transcription factor leads to tumorigenesis. Prognosis of many tumours also depends upon the p53 status. Most of the clinically used anticancer compounds activate p53 dependent pathway of apoptosis and hence require p53 for their mechanism of action. Further, Ras/Raf/MEK/ERK axis is an important signaling pathway activated in many cancers. Dependence of diaminothiazoles, compounds that have gained importance recently due to their anticancer and anti angiogenic activities, were tested in cancer models with varying p53 or Ras/Raf mutational status.

**Methods:**

In this study we have used p53 mutated and knock out colon cancer cells and xenograft tumours to study the role of p53 in apoptosis mediated by diaminothiazoles. Colon cancer cell lines with varying mutational status for Ras or Raf were also used. We have also examined the toxicity and *in vivo* efficacy of a lead diaminothiazole 4-Amino-5-benzoyl-2-(4-methoxy phenylamino)thiazole (DAT1) in colon cancer xenografts.

**Results:**

We have found that DAT1 is active in both *in vitro* and *in vivo* models with nonfunctional p53. Earlier studies have shown that extrinsic pathway plays major role in DAT1 mediated apoptosis. In this study, we have found that DAT1 is causing p53 independent upregulation of the death receptor 5 by activating the Ras/Raf/MEK/ERK signaling pathway both in wild type and p53 suppressed colon cancer cells. These findings are also confirmed by the *in vivo* results. Further, DAT1 is more efficient to induce apoptosis in colon cancer cells with mutated Ras or Raf.

**Conclusions:**

Minimal toxicity in both acute and subacute studies along with the *in vitro* and *in vivo* efficacy of DAT1 in cancers with both wild type and nonfunctional p53 place it as a highly beneficial candidate for cancer chemotherapy. Besides, efficiency in cancer cells with mutations in the Ras oncoprotein or its downstream kinase Raf raise interest in diaminothiazole class of compounds for further follow-up.

**Electronic supplementary material:**

The online version of this article (doi:10.1186/s12943-016-0505-7) contains supplementary material, which is available to authorized users.

## Background

p53 is a tumour suppressor protein that plays a key role in the cellular response to stress and hence acts as a major obstructer of tumorigenesis. Once stimulated by numerous external and internal stress signals, p53 accumulates in the nucleus in its active form leading to either growth arrest or apoptosis. It also contributes to cellular processes such as cellular differentiation, DNA repair and angiogenesis. More than 50 % of human cancers have mutation in the p53 gene which renders it non functional. In many other cancer cases, a loss of p53 function happens through indirect inactivation of the protein when some viral proteins bind to it or due to the mutation of the genes that produce proteins interacting with p53 [1].

*In vitro* and *in vivo* studies have implicated the importance of p53 in apoptosis induced by chemotherapy [2]. Many of the currently used anticancer compounds have a p53 dependent mode of action and most of the cases, p53 acts as a proapoptotic protein. It can be supposed that loss of p53 function can confer resistance to chemotherapy. Indeed, reduced efficacy has been reported for some chemotherapies in tumours with suppressed or mutated p53 [3, 4].

p53 can mediate both extrinsic and intrinsic pathways of apoptosis. Extrinsic pathways of apoptosis is mediated by death receptors belonging to Tumour Necrosis Factor (TNF) super family, finally leading to activation of caspase 8 [5]. On the other hand, mitochondria along with the BCl2 family of proteins play a major role in intrinsic pathway of apoptosis leading to the activation of caspase 9 [6, 7]. The extrinsic pathway can be mediated by p53 through the induction of genes encoding three transmembrane proteins Fas, DR5 and PERP [8–10]. p53 also plays a major role in the intrinsic pathway of apoptosis by the induction of Bax [11], Puma [12], Noxa [13] and APAF-1 [14–16] which facilitate the release of cytochrome c from the mitochondria.

However a large body of evidence suggests that p53 independent apoptotic pathways also occur. It has been shown that in p53 deficient cells, Chk1, Chk2 and ABL upregulates p73 which restores the transactivation of p53 target genes [17, 18]. MAPKs and transciption factors like E2F1, FOXO1 brings about p53 independent activation of caspase 3 in a mitochondria dependent or independent manner [19–21]. p53 independent coupling of DNA damage to caspase 3 activation can also happen via cytosolic translocation of Nur22 which is a nuclear protein [22].

p53 activity is mainly modulated by phosphorylation at different sites and several upstream kinases play major roles in this process. Ras/MAPK pathway has been shown to have role in p53 phosphorylation and modulation in both *in vitro* and *in vivo* models [23]. Cyclin A/B-cdc2 complexes also take part in p53 phosphorylation and hence may also be involved in its stabilization [24].

Diaminothiazoles are a group of antimitotic compounds that inhibit different cancer cell lines by binding to the colchicine binding site of tubulin reversibly [25–27]. They are also effective in multidrug resistant cancers [28]. They are shown to inhibit angiogenesis efficiently [29]. The lead diaminothiazole DAT1 potentiates independent extrinsic pathway activation of apoptosis through upregulation of the death receptor DR5 [30]. DR5 is a member of the TNFR family which contains an extra cellular domain and a domain, which can bind to adaptor moleceules that contain a death domain. This domain then interacts with the initiator caspase 8 to bring about apoptosis. DR5 can initiate apoptosis in a ligand independent manner also [31–33]. Initially DR5 has been shown as a downstream target of p53 [10]. But recently many groups have shown that DR5 can be upregulated independently of p53 [34–37]. In this study, we have investigated the signaling pathways elucidated by a lead diaminothiazole DAT1 *in vitro* and *in vivo*. The toxicity studies have been undertaken. The role of p53 in DAT1 mediated signaling events and apoptosis were studied. We have also investigated the efficiency of DAT1 in cell lines with varying Ras/Raf mutational status.

## Results

### Efficacy of DAT1 in cell lines with nonfunctional p53

Many of the anticancer compounds need p53 for their mechanism of action. However, from the light of numerous studies conducted recently, it can be concluded that correlation of drug sensitivity with p53 status depends very much on the drug and cell type. To test the activity of DAT1 in cell lines where the action of p53 is suppressed, we have used colon cancer cell lines HT29, SW620 and SW480 with mutated p53 [38] and HCT116 p53−/−, an isogenic p53 knock out (p53 −/−) line and compared with p53 wild type cell line HCT116 (p53 +/+). The half inhibitory concentration (IC_50_) of DAT1 was determined in these cell lines by MTT assay and was compared with the widely used chemotherapeutic agents paclitaxel (taxol®), vinblastine and 5-fluorouracil. DAT1 was effective in all these cell lines in a comparable manner irrespective of their p53 status (Table 1). As reported earlier [39–42], taxol maintained its efficacy in cell lines with nonfunctional p53, whereas vinblastine and 5-fluorouracil had decreased efficiency in most of these cell lines.

**Table 1:** Comparison of IC_50_ values of DAT1 with other antimitotic drugs. Cells were treated with the drugs and MTT assay was performed as given in the methods section. Half inhibitory concentration was obtained using the programme Origin

Cell line	DAT1 (μM)	Vinblastine (μM)	Taxol (μM)	5 fluoro uracil (μM)
HCT116	0.3 ± 0.08	0.004 ± 0.002	0.014 ± 0.007	8.69 ± 0.54
HCT116 p53 -/-	0.7091 ± 0.08	0.0195 ± 0.0007	0.006 ± 0.002	19.34 ± 1.34
SW 620	0.42 ± 0.07	0.3 ± 0.09	0.0245 ± 0.006	9.65 ± 0.33
SW 480	0.795 ± 0.09	3.385 ± 0.247	0.0315 ± 0.0014	18.65 ± 0.68
HT 29	0.2 ± 0.08	0.0015 ± 0.0007	0.01 ± 0.0014	20.18 ± 0.77

**Fig. 1:**
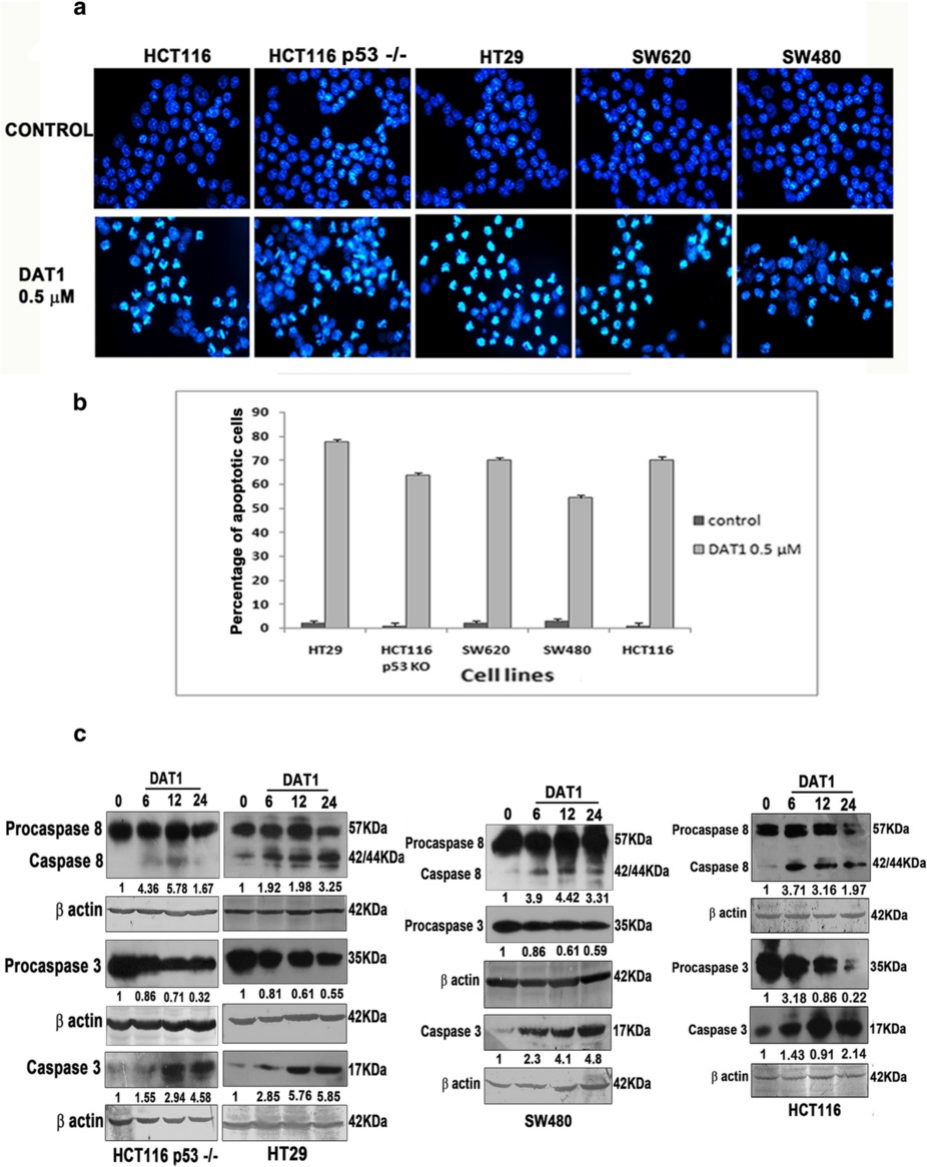
Apoptosis induction by DAT1 in p53 mutated cell lines **a**. Indicated cell lines were treated with DAT1 for a period of 24 h and stained with DAPI. **b**. Apoptosis was quantitated by counting the number of cells with condensed chromosomes and percentage was calculated as compared to the total number of cells. Cells were counted from five different fields. Values expressed in the graph are the average values from three independent experiments **c**. Cells were treated with DAT1 for the indicated time periods and caspase 8 and 3 activation were checked using western blotting. Band intensities determined by Biorad Quantity one software were normalized with the levels of beta actin and fold increase was quantified with respect to the untreated ones

DAT1 was earlier shown to induce apoptosis in different cell lines [30]. Treatment of DAT1 to the cell lines with nonfunctional p53 followed by staining with the DNA binding dye DAPI detected condensation of chromosomes (Fig. 1a) which is a hallmark of apoptosis. Quantitation of apoptosis showed that DAT1 was quite effective in all these cell lines (Fig. 1b). The effector caspase 3 activation was also observed in these cell lines (Fig. 1c). However, another microtubule targeting anticancer drug vinblastine, failed to induce apoptosis in p53 knock out (p53 −/−) line at a concentration of 0.01 μM, a concentration in which it displayed large apoptotic response in the p53 wild type HCT116 cells (Additional file 1: Figure S1).

A number of earlier studies indicate that p53 is crucial in the mitochondria mediated apoptotic pathway. However, the extrinsic pathway can be both p53 dependent or independent. Previous studies from our laboratory showed the involvement of both apoptotic pathways in DAT1 mediated apoptosis with major contribution from an independent extrinsic pathway. Since DAT1 was also found to be highly efficient in cells where the intrinsic pathway was blocked [30], it seemed worthwhile to analyze whether the extrinsic pathway of apoptosis caused by DAT1 would be p53 mediated.

Western blotting of lysates prepared from cells treated with DAT1 for different time periods showed that DAT1 caused caspase 8 activation in cell lines irrespective of their p53 status (Fig. 1c).

### p53 independent activation of DR5 by DAT1

**Fig. 2:**
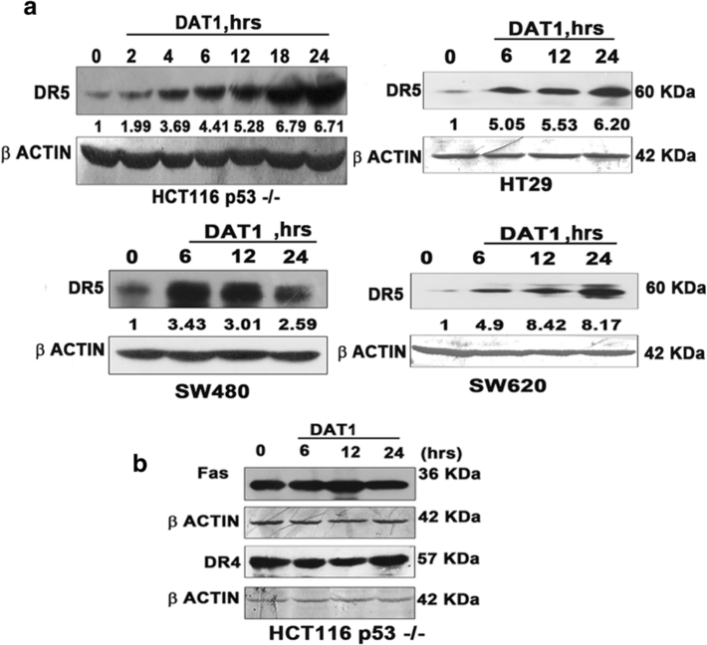
DR5 induction by DAT1 in p53 mutated cell lines **a**. HCT116 p53 −/−, SW620, SW480 and HT29 were treated with DAT1 at ~1.5 X IC_50_ concentration for the indicated time periods and DR5 activation was checked by western blotting. **b**. HCT116 p53 −/− was treated with DAT1 and western blotting was performed using DR4 and Fas antibodies

Several downstream targets of p53 are involved in apoptosis. Bax, PIGs (p53 induced genes), Fas/APO-1, DR5 are some of the downstream targets of apoptosis that play major roles in apoptosis [34]. Previous studies from our laboratory have shown that DAT1 mediated apoptosis depends mainly on the DR5 activation [30]. DR5 is a member of the TNFR family and is homologous to the other death receptor DR4 [43]. Both DR4 and DR5 gets activated when bound by its substrate TRAIL and recruits Fas associated death domain (FADD) which in turn recruits caspase 8 and initiates extrinsic pathway of apoptosis. DAT1 treatment caused DR5 upregulation in cells with non functional p53 confirming that DAT1 mediated DR5 induction is p53 independent (Fig. 2a). There was no considerable change in the levels of DR4 and the other member of the TNF receptor family, Fas receptor, after DAT1 treatment (Fig. 2b).

### ERK activation and nuclear translocation

**Fig. 3:**
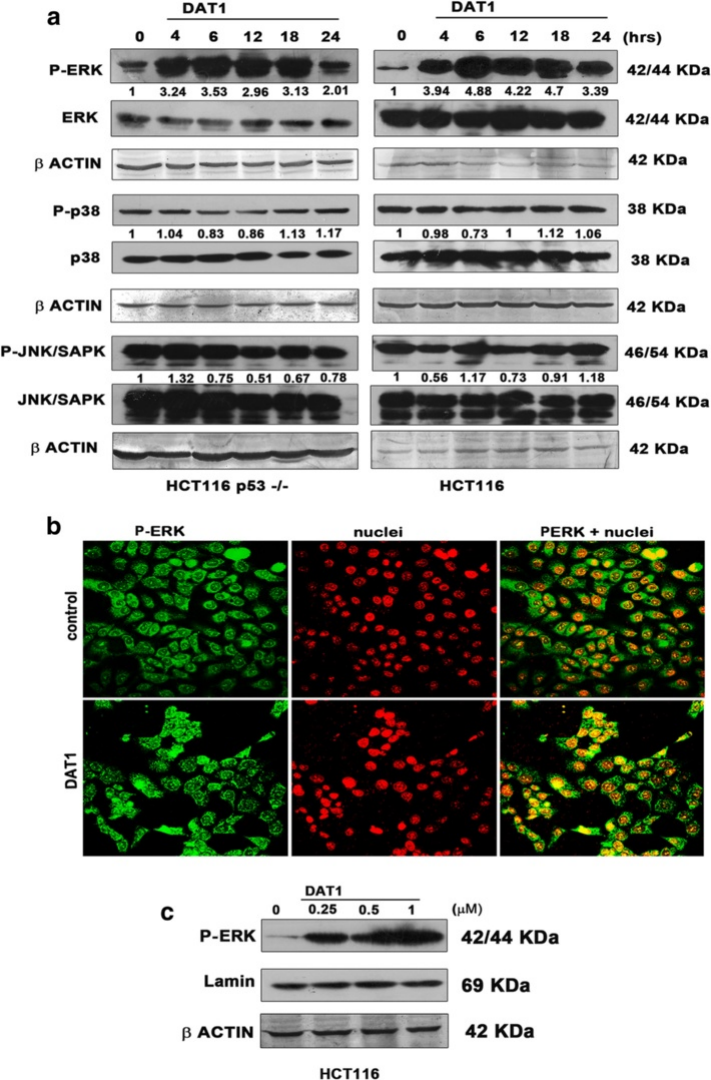
ERK activation and its nuclear translocation by DAT1 **a**. Cells were treated with DAT1 for the indicated time periods and immunoblotted with the respective antibodies. Band intensities of P-p38, P-ERK and P-SAPK/JNK were quantified and were normalized with the levels of p38, ERK and SAPK/JNK respectively. **b**. Nuclear translocation of ERK was checked in HCT116 cells by immunofluoresence. Cells were treated with DAT1 for 24 h, fixed and probed with P-ERK antibody followed by alexaflour 488. Nuclei were stained with propidium iodide and imaged using a confocal microscope. **c**. Nuclear extract was collected from HCT116 cells and immunoblotting was performed using P-ERK antibody. Lamin was used as the nuclear loading control

Mitogen activated protein kinases are serine/threonine specific protein kinases and comprises of Extracellular signal Regulated stress Kinases (ERK), c-Jun N-terminal kinase/stress activated protein kinases (JNK) and p38 kinases [44, 45]. Among the many isoforms, ERK1/2 is the most extensively studied isoform and is activated by phosphorylation in response to various stress stimuli through diverse mechanisms involving the Ras-Raf-MEK pathway. A variety of biological responses (apoptosis, proliferation, migration and differentiation) are associated with ERK activation depending on the cell type, duration of activation and stimulus [46, 47]. Thus we were interested to know whether MAPK signaling has any role in the DAT1 mediated apoptosis. Both HCT116 and HCT116 p53−/−cells were treated with DAT1 for the indicated time periods, and P-ERK, P-p38, P-JNK levels were checked. DAT1 caused P-ERK activation in HCT116 and HCT116 p53−/− cells, while P-p38 and P-JNK levels remained same (Fig. 3a). Further, immunofluorescence and western blotting experiments also revealed increase in nuclear localization of P-ERK following DAT1 treatment (Fig. 3b, c) indicating an increase in their role for transcription of molecules involved in further cellular processes.

### Decrease in apoptosis upon blocking ERK activation

**Fig. 4:**
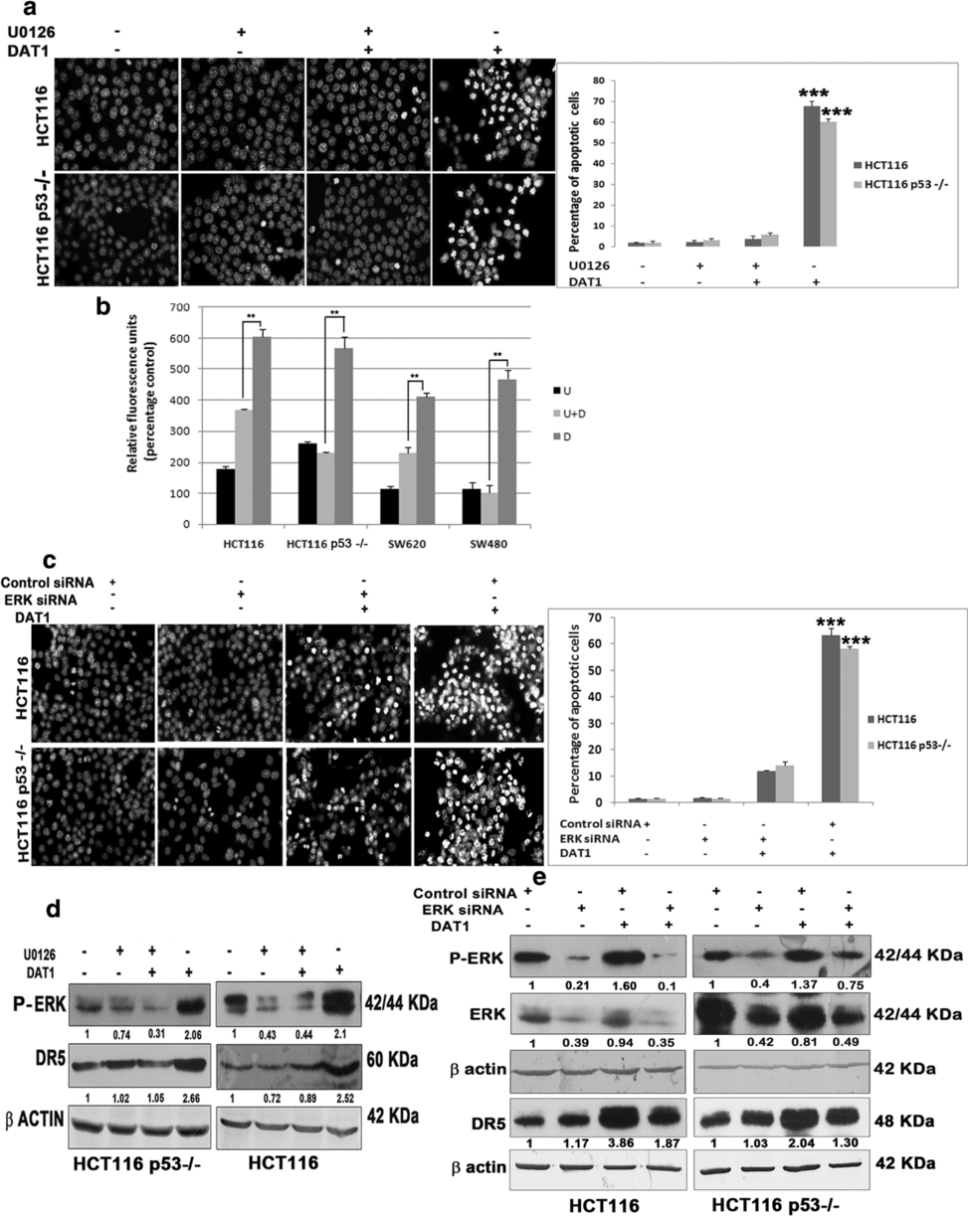
Apoptosis by DAT1 upon blocking ERK activation **a**. Cells were treated with DAT1 in the presence or absence of the MEK inhibitor U0126. Subsequently, they were fixed and stained with DAPI and imaged in a fluorescence microscope. Percentage of apoptosis was calculated and plotted as given in Fig. 1. *** denotes *p* ≤ 0.001 and indicates that the difference between the percentage of apoptosis caused by DAT1 in presence and absence of the inhibitor is extremely significant. **b** Cells were treated with DAT1 for 24 h with or without the presence of the MEK inhibitor and cell lysates were collected. Caspase 3 assay was performed according to manufacturer’s instructions and analysed as given in the methods section. ** for *p* ≤ 0.01 and indicates significant difference in the fluorescence intensities arising due to caspase 3 activation in the presence and absence of the inhibitor. **c**. ERK was inhibited by siRNA against ERK, followed by DAPI staining in the presence or absence of DAT1 and the percentage of apoptosis was calculated and plotted. **d**,**e**. Lack of DR5 activation upon blocking ERK activation was checked by western blotting. Indicated cell lines were treated with DAT1 for a period of 18 h in the presence or absence of the inhibitor (**d**) or in the presence or absence of ERK siRNA (**e**)

U0126 is a selective inhibitor of MEK1/2 and hence prevents ERK1/2 activation by its phosphorylation. HCT116 and HCT116 p53−/−cells were treated with U0126 followed by DAT1 treatment and the chromatin condensation was checked by DAPI staining. It was seen that the percentage of apoptotic cells decreased from 67 to 3 % and 60 to 5 % in HCT116 and HCT116 p53 −/− respectively in the inhibitor treated cells, as compared to cells treated with DAT1 alone (Fig. 4a). The reduction of apoptosis was also observed by spectrofluorometric measurement of active caspase 3 in cells treated with DAT1 either in the presence or absence of the ERK inhibitor U0126. The relative fluorescence intensity (which indicates caspase 3 activation) decreased in the presence of U0126 as compared to treatment with DAT1 alone (Fig. 4b). The findings were also supported using RNAi inhibition of ERK1/2. Prominent reduction of apoptosis as measured by chromatin condensation was observed using siRNA against ERK1/2 as compared to scrambled siRNA (Fig. 4c).

### Lack of induction of DR5 by ERK inhibition

There are many studies which show that ERK signaling can be an upstream regulator of DR5 activation [48–50]. To check whether there is any link between ERK activation and DR5 upregulation in DAT1 mediated apoptosis, we tested for DR5 induction following DAT1 treatment either in the presence or absence of U0126 in HCT116 and HCT116 p53−/− cells. DR5 induction by DAT1 was significantly hampered in presence of U0126 (Fig. 4d) proving that DAT1 induces ERK mediated DR5 activation. Reduced DR5 induction was also observed using siRNA against ERK1/2 as compared to scrambled siRNA (Fig. 4e).

Thus ERK activation was found to be a key signaling mechanism upstream DR5 mediated apoptosis by DAT1 in colon cancer cells with both wild type and nonfunctional p53.

**Fig. 5:**
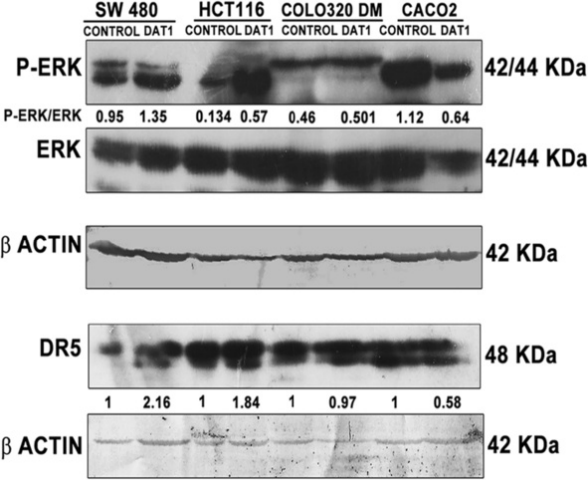
Comparison of ERK activation in cell lines of differential Ras/Raf mutational status. SW480, HCT116, Colo320 DM and Caco 2 cell lines were treated with DAT1 (~1.5 times IC_50_ concentrations). ERK or DR5 activation was checked by western blotting after probing with the respective antibodies. The band intensities were normalized to the levels of actin and P-ERK/ERK ratios were found out

Ras/Raf/MEK pathway plays an important role upstream of ERK kinase activation. Mutations in the several isoforms of the small GTPase Ras or Raf kinase superfamily of proteins were found to be associated with different types of human cancers [51, 52]. We thus wanted to see the effect of Ras/Raf mutation on the signaling and DAT1 activity in colon cancer cell lines. The IC_50_ values of DAT1 and Ras/Raf mutational status in five different cell lines are shown in Table 2. The apoptosis induction, as measured by chromosome condensation, was also done in the cell lines Colo 320 DM and Caco2 (Additional file 2: Figure S2) with wild type Ras/Raf. It was observed that at approximately 1.5 times the IC_50_ concentration, DAT1 was able to induce only 38 and 32 % apoptosis respectively in these cells (Fig. 1 for comparison with other cell lines). Further, as shown in Fig. 5, DAT1 was found to activate ERK in SW 480 and HCT116 cell lines where Ras mutations [53–55] were noted but it was unable to activate ERK in Colo 320 DM or Caco2 cell lines with wild type Ras or Raf [54, 56, 57]. Consequently, DR5 was also found to be upregulated upon DAT1 treatment in SW480 and HCT116 cells but not in Colo320 DM or Caco2 cells showing that DAT1 was more effective to induce apoptosis by modulating Ras/Raf/MEK/ERK mediated death receptor pathway in cells with Ras/Raf mutation.

**Table 2:** Comparison of IC_50_ values of DAT1 in Ras/Raf mutated and wild type cell lines. The respective cell lines were treated with DAT1 for 48 h at different concentrations followed by MTT assay. IC_50_ values were calculated using the programme Origin

Cell lines	Ras/Raf mutation status [53, 54, 64]	IC_50_(μM)
SW 480	Ras mutated,Raf wild type	0.795 ± 0.09
HT 29	Raf mutated,Ras wild type	0.2 ± 0.08
HCT116	Ras mutated,Raf wild type	0.3 ± 0.08
Colo 320 DM	Wild type Ras and Raf	0.6546 ± 0.048
Caco 2	Wild type Ras and Raf	5.315 ± 0.1622

We further continued to check the efficacy of DAT1 *in vivo* using colon cancer xenografts and the signaling and apoptotic mechanisms therein.

### In vivo efficacy of DAT1

**Fig. 6:**
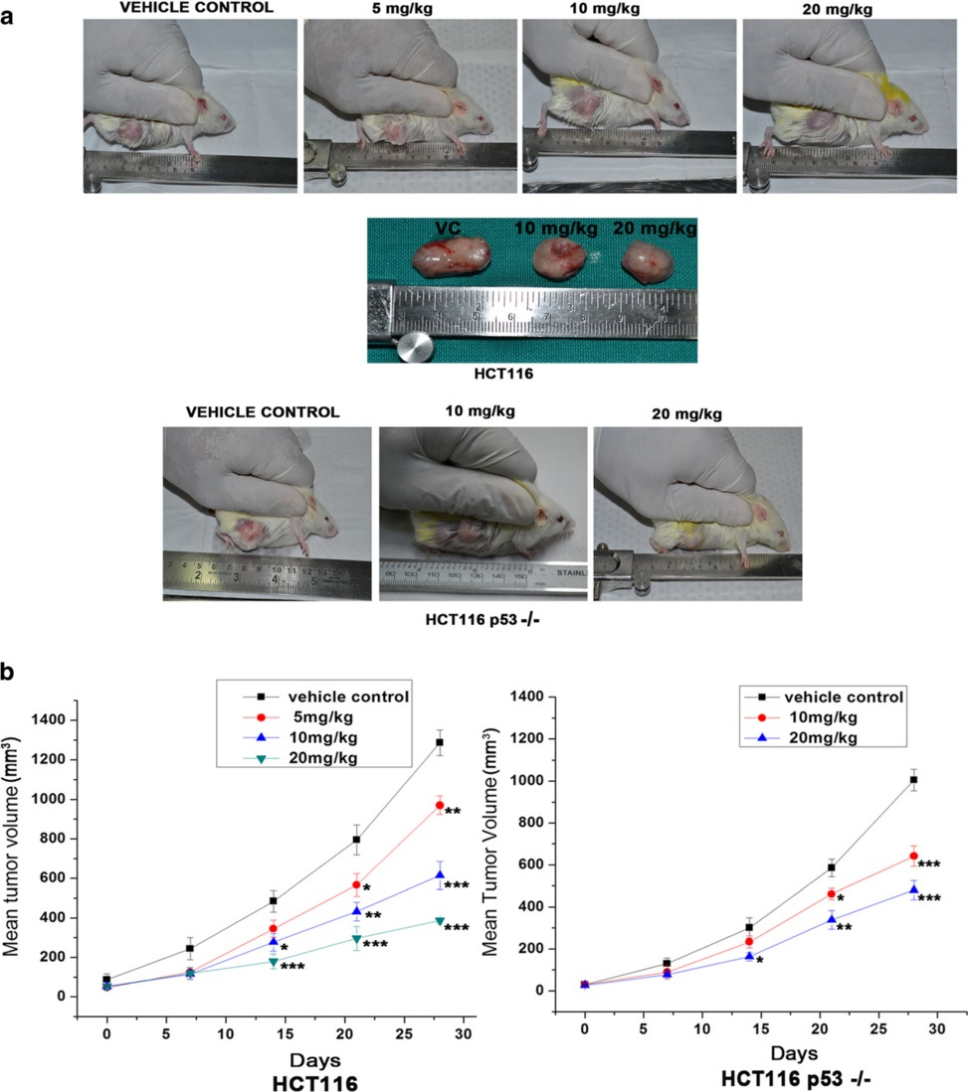
Effect of DAT1 on colon cancer xenografts. HCT116/HCT116 p53 −/− cells were injected subcutaneously into SCID/NOD mice. Once the tumour volume reached about 20–80 mm^3^, DAT1 was administered intraperitoneally. **a**. Photographs of SCID mice showing decreased tumour size upon DAT1 treatment. **b**. Tumour volumes were measured weekly both in control and the treated tissues and were plotted against the number of days. The statistical significance of differences in tumour volumes upon treatment are denoted as * for *p* ≤ 0.05, ** for *p* ≤ 0.01 and *** for *p* ≤ 0.001

Tumours were grown in Severe Combined immunodeficiency/Non obese diabetic (SCID/NOD) mice by injecting HCT116 or HCT116 p53 −/− cells subcutaneously. As shown in Fig. 6, DAT1 treatment for 28 days reduced the mean tumour volume effectively in a concentration dependent way for both HCT116 and HCT116 p53−/− xenograft models.

### Acute and Sub- acute toxicity studies of DAT1

To rule out the possibility that the efficacy of DAT1 in tumours is not due to toxicity of the compound in mice, we determined both the acute and sub-acute toxicity of DAT1 in Swiss Albino mice for the range of concentrations used for xenograft studies. In acute toxicity studies, where single doses were administered to different groups, there were no signs of weight loss, dehydration, mortality or behavioural change after 14 days upto 50 mg/kg of DAT1 (Additional file 3: Table S1).

In sub-acute toxicity studies, animals were administered with 4, 8 or 16 mg/kg of DAT1 on alternate days for the first 10 days and twice weekly thereafter for a period of 3 months and then compared with the control group of animals. At the end of 3 months, there was no significant change in the weight. Further, as shown in Table 3, hematological analysis detected no significant change in the RBC and WBC content of the treated mice from the control mice indicating that there was no hematotoxicity. The serum AST, ALP, BUN and creatinine levels of the treated mice also did not vary much from the control values indicating that the drug did not induce hepato and renal toxicity (Table 3). The serum ALT level remained similar to the control for the groups that were administered with 4 or 8 mg/kg DAT1. However, the ALT level increased for mice that were administered 16 mg/kg of DAT1. But the increase could be attributed to solvent toxicity as the vehicle control containing similar amount of cremaphor/ethanol showed similar toxicity for ALT.

**Table 3:** Analysis of blood parameters upon DAT1 treatment. Swiss Albino mice were injected with DAT1 as given in the methods section. After 3 months, serum ALP, ALT, AST, BUN and creatinine levels were determined according to manufacturer’s protocol. WBC and RBC count was taken using hemocytometer. Group I: Control, Group II: 4 mg/kg, Group III: 8 mg/kg, Group IV: 16 mg/kg and Group V: vehicle control

Groups	ALT (U/L)	ALP (U/L)	BUN (mg/dL)	AST (U/L)	Creatinine (mg/dL)	RBC (million/mm^3^)	WBC (per mm^3^)
Group I	46.45 ± 8.1	193.64 ± 10.1	24.16 ± 5.28	123.84 ± 26.14	0.743 ± 0.06	4.9 ± 0.52	7500 ± 1742
Group II	54.38 ± 4.15	158.32 ± 8.32	29.8 ± 1.25	128 ± 25.1	0.82 ± 0.1	4.42 ± 0.75	8100 ± 1979
Group III	53.845 ± 4.81	136.48 ± 10.69	29.8 ± 1.26	128.165 ± 1.87	0.76 ± 0.07	4.48 ± 0.34	6220 ± 1626.34
Group IV	148.39 ± 14.55	155.61 ± 15.15	29.8 ± 1.27	162.67 ± 12.73	0.58 ± 0.09	4.475 ± 0.26	4550 ± 264.575
Group V	140.64 ± 8.59	198.51 ± 10.1	29.8 ± 1.28	167.67 ± 10.73	0.658 ± 0.042	5.08 ± 0.438	7267 ± 141.42

### Induction of apoptosis in the tumour tissues

**Fig. 7:**
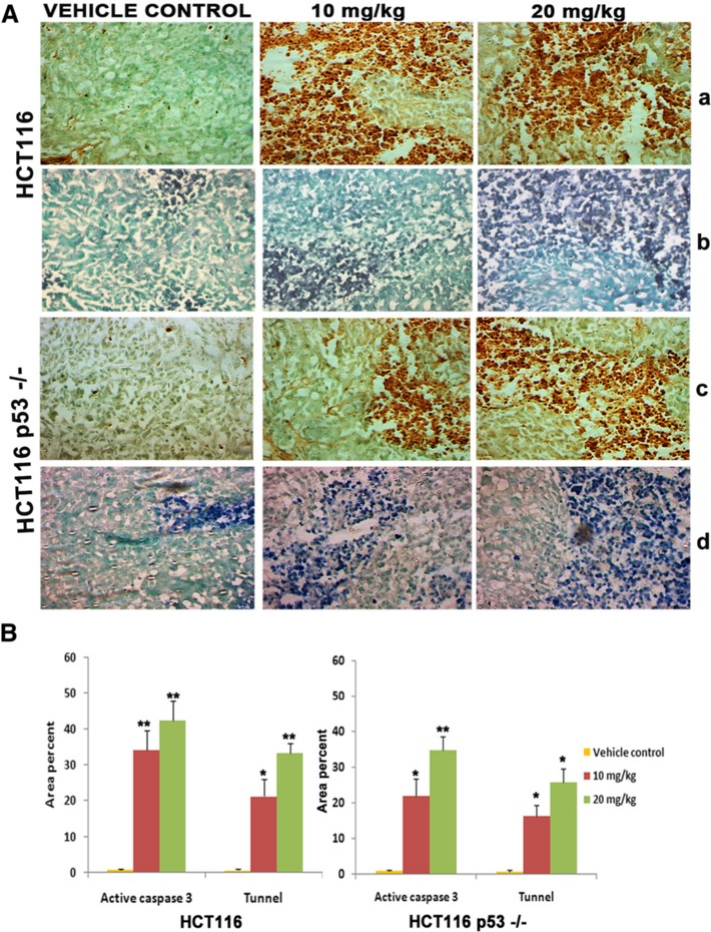
Measurement of apoptosis in tumour tissues. **A**. Tumours bearing HCT116 or HCT116 p53 −/− cells were excised after 28 days of injection, fixed in 4 % paraformaldehyde and preserved in 30 % sucrose and cryosectioning was performed. **a**,**c**. Tunnel assay. Brown color (DAB staining) indicates the apoptotic cells. **b**,**d**. Immunuhistochemical staining of active caspase 3 in HCT116 and HCT116 p53 −/− . Alkaline phophatase detection method was used to visualize the antibody bound cells. **B**. The area showing blue/brown colour were quantified by Image J software and plotted. *p* values for the differences among treatment values and control values were obtained by unpaired *t* test,** stands for *p* ≤ 0.001 and * for *p* ≤ 0.01. At least five fields were analysed

We further checked whether the reduction of tumour sizes were due to apoptosis induction by DAT1. Apoptosis induction in the tumour tissues was analyzed by tunnel assay. As seen in (Fig. 7), there were much more staining in the treated tissues as compared to the control tissues indicating prominent apoptosis in DAT1 treated colon xenografts with both wild type and nonfunctional p53. This was also confirmed by immunohistochemistry, where the tissues were probed with active caspase 3 antibody. Here also, more cells showed positive staining for active caspase 3 in the treated tissues as compared to the control tissues (Fig. 7) confirming more apoptosis in the treated tissues.

### Activation of DR5 and P-ERK in the tumour tissues

**Fig. 8:**
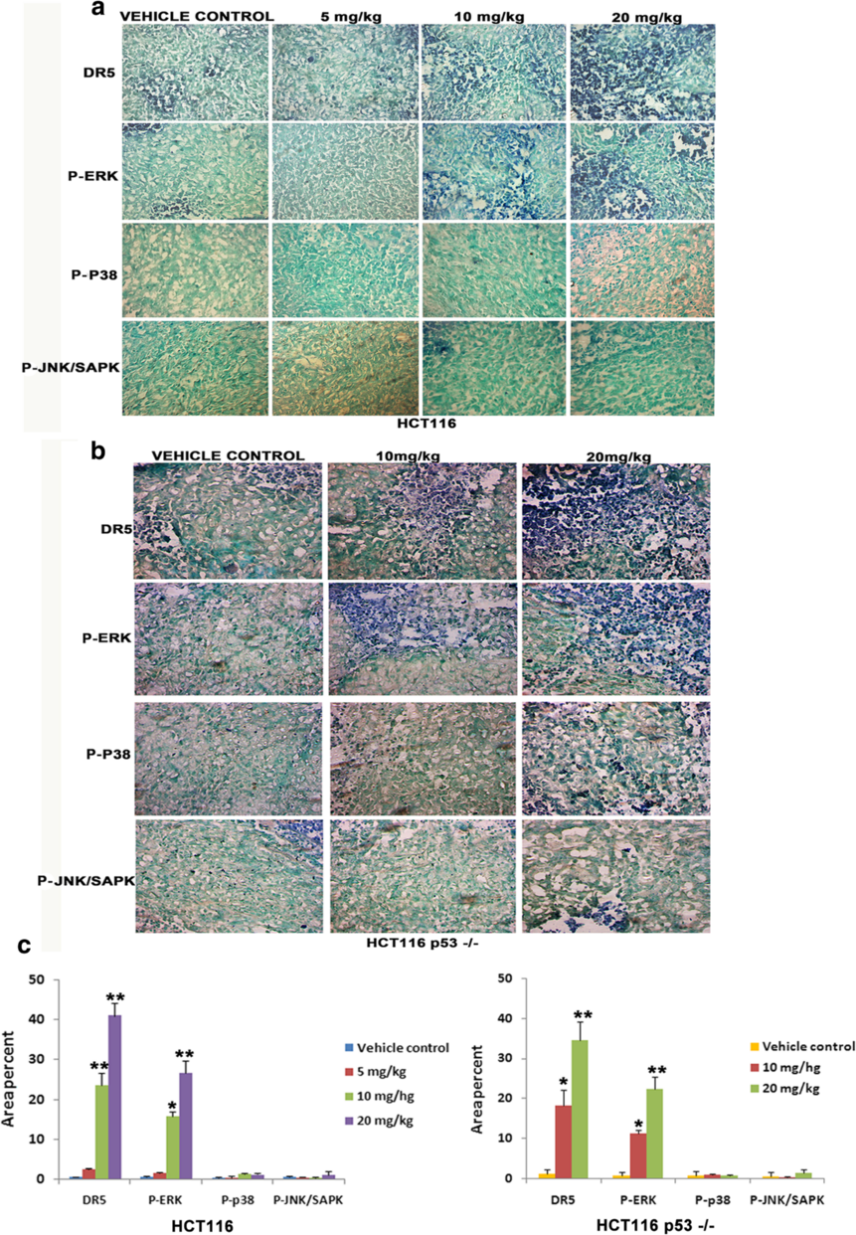
Signaling pathway elucidated by DAT1 in tumour tissues. **a**-**b**. Sections were made from control and DAT1 treated tumour tissues and immunohistochemistry was performed using the respective antibodies as described in Fig. 7. **c** The positively stained area were quantified using Image J and were plotted to show activation of the respective proteins followed by determination of statistical significance using unpaired *t* test

Experiments in colon cancer cell lines have shown that DAT1 is causing DR5 and P-ERK activation. In order to confirm these observations *in vivo*, DR5, P-ERK, P-JNK/SAPK and P-p38 levels were checked in tissues from both p53 wild type and knock out mice by immunohistochemistry. Clearly, as indicated by the increase in blue staining, DR5 and P-ERK levels increased in the treated tissues as compared to the control tissues showing activation of the respective proteins by DAT1 (Fig. 8a -b). However, consistent with the *in vitro* results, the tissues labeled with P-p38 and P-JNK/SAPK antibodies displayed similar staining pattern in both treated and control tissues showing that these pathways were not activated by DAT1 in colon tumours.

**Fig. 9:**
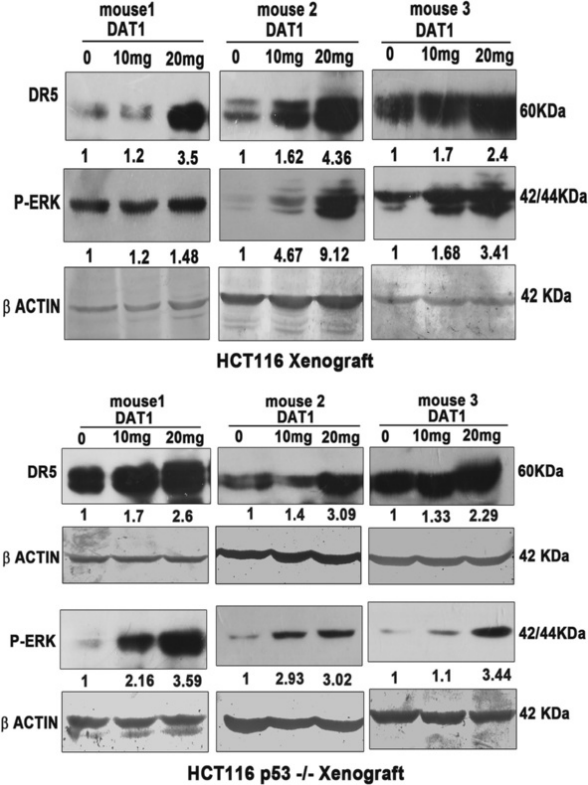
ERK and DR5 activation in the tumour tissues detected by immunoblotting. Western blots showing activation of DR5 and pERK in the treated tissues. Tissue lysates were analyzed by SDS-PAGE and western blotting was performed with the respective antibodies as shown. Blots were developed by chemiluminescence

DR5 and P-ERK activation was also confirmed by western blotting. Blots from three different mice are shown in Fig. 9. The levels of DR5 and P-ERK were found to be more in the treated tissues as compared to the control tissues, thus supporting our findings from cell line studies.

## Discussion

The protein p53 is involved in transcription or suppression of many genes that are involved in apoptosis, autophagy, cellular differentiation and angiogenesis. It protects the cells from many stresses that may give rise to cancer and it has been found that cancers with nonfunctional p53 are difficult to control and treat. Thus our findings in this study that the diaminothiazole DAT1 is effective in colon cancer cells and tumour xenografts even with nonfunctional p53 are important.

DAT1 mediated apoptosis was earlier shown to be triggered by both mitochondria mediated and death receptor mediated pathways. It was also shown that unlike many other drugs, the extrinsic or death receptor pathway triggered by DAT1 in colon cancer cells was independent of the intrinsic pathway and played the major role in inducing apoptosis [30]. p53 is almost always linked with the intrinsic pathway whereas there are various reports that show that the extrinsic pathway can be both p53 dependent or independent depending on the nature of the stress and environment. Since DAT1 was active even in cells where the intrinsic pathway was blocked, we were interested to study whether it would be active in cancers with nonfunctional p53. From *in vitro* and *in vivo* studies, we have found that DAT1 is effective both in cell lines and tumours with wild type or nonfunctional p53 status. In the *in vivo* studies, our results show that the efficiency was about 19 % less (53.4 % vs. 72.3 % inhibition) in case of tumours bearing HCT116 p53 −/− cells as compared to HCT116 cells for DAT1 treatment of 20 mg/kg (Fig. 6b). The somewhat reduced efficacy in the later case is possibly due to the involvement of p53 in the less prominent intrinsic pathway of apoptosis induced by DAT1. A confirmation of this hypothesis came from the fact that DAT1 induced the phosphorylation and nuclear localization of p53 in HCT 116 cells (Additional file 4: Figure S3).

In this report we have found that DAT1 can trigger death receptor 5 independent of p53. p53 independent upregulation of DR5 has been reported earlier in certain cases. Urasolic acid, a triterpene, was shown to upregulate DR4 and DR5 independent of p53 through the involvement of reactive oxygen species and JNK [35]. Proteasome inhibitors and TRAIL receptor expression by glucocorticoids and interferon-gamma were also reported to have p53-independent upregulation of DR5 [34, 36]. Upstream activation of ERK but not the other map kinases was found to be a regulator of DR5 upregulation in DAT1 induced apoptosis in colon cancer as both inhibition of MEK by the selective inhibitor U0126 and small RNA mediated downregulation of ERK significantly reduced DR5 activation and DAT1 mediated apoptosis. This is in accordance with earlier reports that link extra cellular signal regulated kinase pathway and DR5 activation [48–50]. Paclitaxel (Taxol), another antimitotic drug that is widely used in clinics, also has been reported to strongly activate ERK and P-p38 in breast cancer cells and induce apoptosis in a p53 independent way [40]. Whether the efficacy of taxol and DAT1 in p53 defective tumours through ERK and DR5 activation is related to their antimicrotubular property, is not known at this point.

Furthermore, DAT1 was found to be more active in inducing apoptosis in colon cancer cell lines where the small GTPase protein Ras or its downstream kinase Raf were mutated. This could be because in these cell lines, it efficiently activates ERK followed by DR5 as opposed to cells where Ras or Raf kinase were of wild type. In the later cases, the limited apoptosis by DAT1 could be induced only by the intrinsic pathway and the cell death might also be contributed by some other death mechanisms.

Ras/Raf/MEK/ERK cascade is an important signaling pathway in cancers and activation of this pathway is shown to play role in both cell proliferation and apoptosis [58, 59]. Mutations in Ras or Raf are known to induce constitutive activation of ERK which further act as a transcription factor to many prosurvival or pro apoptotic proteins. In our experiments, however, we did not get more constitutive activity of ERK in HCT116, HT29 (data not shown) or SW480 cells with mutated Ras or Raf as compared to Colo320DM or Caco2 cells where these proteins were reported to be of wild type (Fig. 5). There is however a report that higher expression of the mitogen-activated protein kinase phosphatase-1 (MKP1) may inactivate ERK in HCT116 cells as opposed to Caco2 cells where MKP1 expression is low giving rise to sustained ERK activation [60]. About 33 % of cancers have mutations in Ras and about 8 % harbor mutations in Raf. Considering the high importance of this pathway in different types of cancer, inhibitors are now in clinical trials [61, 62] . However, these inhibitors target the prosurvival role of this pathway activation. Considering this, role of DAT1 to induce the proapoptotic function of this pathway would be of high interest and it provokes our quest to know the fine tuning of this pathway for the initiation of survival response or apoptotic response.

## Conclusions

Thus, the diaminothiazole DAT1 is able to induce apoptosis in both *in vitro* and *in vivo* colon cancer models overcoming the lack of p53 functionality through ERK mediated upregulation of Death Receptor 5. Further, DAT1 is more effective to induce apoptosis in cell lines with mutated Ras oncoprotein or its downstream kinase Raf. These findings, along with its minimal toxicity in both acute and sub-acute studies, place diaminothiazoles as highly beneficial candidates for cancer chemotherapy and call for the need of clinical trials.

## Methods

### Materials

Cell lines HCT116 and SW480 were from ATCC. SW620 and HT29 were from NCI. HCT116 p53 −/− was a kind gift from Dr. Bert Vogelstein. Caco2 and Colo 320 DM cell lines were procured from the national repository of National Centre for Cell Sciences, Pune, India. DAT1 was synthesized by slight modification of the method described earlier [63]. Vinblastine, taxol, 5-Fluoro uracil, DAPI, propidium iodide and U0126 were from Sigma, USA. siRNA construct for ERK1/2 was obtained from Santacruz Biotechnology. DMEM and PSN antibiotic mixture were purchased from Invitrogen, USA. MTT and Caspase 3 fluorogenic substrate were from USB and BD pharmingen, respectively. Tunnel assay kit was from R & D Biosystems, USA and immunohistochemical kit was purchased from Vector Labs, USA. Kits for detection of ALP, AST, ALT, Creatinine and BUN levels in serum were from Aspen Laboratories, India.

### Antibodies

DR5 antibody was from Imgenex, Canada or Abcam, UK, DR4 antibodies were procured from Imgenex, Canada. Antibody against Fas was from Sigma and antibodies against P-ERK, P-p38, P-JNK/SAPK, Caspase 3 and 8 were from Cell Signaling Technologies, USA or Abcam, UK. Lamin, β actin, ERK, p38 and JNK, SAPK antibodies were purchased from Santacruz Biotechnology, USA. Alexafluor 488, 568 and 633 antibodies were from Molecular Probes (Invitrogen). Anti rat tubulin marker antibody was from Abcam, UK.

### Maintenance of cell lines

The cell lines used in the study were maintained in DMEM or RPM1 containing 10 % FBS with 1X Penicillin Streptomycin Neomycin antibiotic mixture. The cells were incubated at 37 °C in a CO_2_ incubator in humid condition containing 5 % CO_2._ From the reference stock, frozen stocks of cells were made within passage 3 and stored in liquid nitrogen. For experiments, cells were used within 3 months after revival.

### DAPI staining for detection of apoptosis

The cells were grown on coverslips in 24 well plates and incubated with respective drug or inhibitor for 24 h. They were then washed with PBS, fixed with chilled methanol-EDTA for 10 min and rehydrated with PBS for 10 min at room temperature. Successively, the cells were treated with DAPI (0.5 μg/ml) for 2 min in the dark. The coverslips were then mounted on fluoromount G and viewed at 40X in the UV region using a fluorescence microscope (Olympus IX71).

### Western blotting

Cells were grown to 70–80 % confluency in 60 mm culture dishes. After drug treatment, they were harvested and lysed. Protein samples from tissues were collected by homogenizing the tissue in the presence of lysis buffer, followed by centrifugation for 20 min. The protein samples were run in a 10–12 % gel and transferred on to a PVDF membrane. The membranes were probed with specific primary antibodies (1:1000) overnight followed by secondary antibodies conjugated with horse radish peroxidase/alkaline phosphatase (1:2000) for 1 hr at room temperature and were developed by chemiluminescence or alkaline phosphatase method.

### Cytotoxicity assay

Cells were seeded in 96 well plates. At about70 % confluency, they were treated with different concentrations of drugs either individually or in combination. After 48 h, MTT assay was done (Mossmann 1983) and absorbance was measured at 570 nm (Biorad Microplate reader). Percentage viability was plotted against the respective concentrations of the drugs and half inhibitory concentration (IC_50_) was calculated with the non linear regression programme of Origin.

### Immunofluorescence

Cells were seeded on coverslips in 35 mm dishes and were treated with DAT1 for the indicated time period. After fixing with methanol-EDTA and blocking, they were probed with the primary antibodies overnight followed by alexafluor 488. Nuclei were stained with propidium iodide and were imaged in a confocal microscope (Leica SP2) or in Olympus IX71.

### Caspase 3 fluorometric assay

The enzymatic activities of caspase 3 was assayed spectrofluorimetrically using fluorogenic substrates from BD Pharmingen according to manufacturer’s instructions and then quantitated using a fluorescence plate reader (Tecan Infinite M200) with the excitation and emission wavelengths of 400 and 505 nm, respectively. The values of relative fluorescence units with respect to the controls were plotted against different treatments.

### Extraction of nuclear and cytoplasmic fractions

10^5^ cells were treated with DAT1 for the indicated time period after attachment. Following incubation, 0.5 mL Buffer A (10 mM HEPES, pH 7.9, 10 mM KCl, 0.1 mM EDTA, protease inhibitors) was added, and cells were collected by scraping. After centrifugation at 13000 rpm for 3 min, the supernatant was preserved as cytoplasmic extract. To the pellet 150 μL of Buffer B (20 mM HEPES, pH 7.9, 0.4 M NaCl,1 mM EDTA, 10 % Glycerol) was added and kept in ice for 1 h with intermittent shaking followed by centrifugation at high speed. The supernatant was collected as the nuclear extract.

### siRNA transfection

Cells were seeded in 35 mm dishes and after attachment, they were transfected with ERK or control siRNA using the transfection reagent lipofectamine 2000. The cells were further incubated for 72 h before addition of drug and processing for western blotting or DAPI staining.

### Animal experiments

Swiss Albino mice and SCID/NOD mice were used for the experiments. Animals weighing 25–30 g and age of 9–11 weeks were used in the experiments. All the animal experiments were conducted as per the approved guidelines of Institute Animal Ethics Committee which is under the Committee for the Purpose of Control and Supervision of Experiments on Animals (CPCSEA), Govt. of India. The mice were handled and housed in conventional plastic cages and maintained on an automatic 12 h lighting cycle at a temperature of 22-24 °C.

### Acute toxicity studies

In acute toxicity studies, Swiss Albino mice were divided in to six groups of six animal each and DAT1 (12–50 mg/kg) was administered intraperitoneally (i.p) and observed for 14 days for signs of weight loss, dehydration or mortality. DAT1 was dissolved in vehicle cremaphor:ethanol (1:1) and diluted in 0.9 % saline.

### Sub-acute toxicity studies

In sub-acute toxicity studies, Swiss albino mice were divided into five groups of eight animals each. Three groups of Swiss Albino mice were administered (i.p injection) with 4, 8 or 16 mg/kg DAT1, and the control and vehicle control groups were administered with 0.9 % saline or vehicle respectively. Injections were given every alternate day for the first 10 days and after that, twice weekly for a period of 3 months. After 3 months animals were sacrificed and whole blood, liver and kidney were collected. Hematotoxicity was checked by RBC and WBC count. Hepatotoxicity was measured by monitoring the serum ALT and AST levels. Nephrotoxicity was measured by monitoring BUN and creatinine levels in the serum. ALP activity was measured for detection of liver or bone toxicity.

### Xenograft studies

SCID/NOD mice were divided into groups of ten and allowed to acclimatize. After 5 days, tumours were raised by injecting HCT116 or HCT116 p53 −/− cells (1.5 × 10^5^ cells suspended in PBS) subcutaneously in the inner flank region. Once the tumour volumes reached 20–80 mm^3^, drug was administered intraperitoneally, thrice a week at different concentrations.

#### Immunohistochemistry

Tumour tissues from the treated and control mice were collected after sacrificing the mice and fixed in 4 % paraformaldehyde and kept at 4 °C for 12 h. Tissues were then stored in 30 % sucrose until cryo-sectioning. Tissues were sectioned in a Leica CM1850uv cryostat and then processed for immunohistochemistry according to the manufacturer’s protocol (Vector Labs) using the appropriate antibodies. The antibody bound area was visualized using alkaline phosphatase detection method.

### Tunnel assay

Fixed tissues were stored in sucrose and sectioned in a cryostat. The sections were collected in gelatin coated slides and tunnel assay was done according to manufacturer’s protocol (R & D Biosystems, USA). Diaminobenzidine (DAB) staining was used to detect the apoptotic cells.

### Analysis of data

The number of apoptotic cells was determined by counting from at least five fields. Results are shown as mean ± standard deviation. Standard deviations were determined from three independent experiments. Biorad quantity one software was used for densitometric analysis of blots. Blots were normalized with actin, ERK, p38 or JNK/SAPK wherever necessary. For animal experiments, results are shown as mean ± standard error of mean. Data for at least six animals for sub-acute toxicity studies and eight animals for xenograft studies were analyzed. Unpaired student’s *t* test was used for finding the statistical significance and *p* ≤ 0.05 were considered as statistically significant.

## Additional files

Additional file 1: Figure S1. Comparison of apoptosis in HCT116 and HCT116 p53 −/− cells treated with vinblastine : Cells were treated with vinblastine (0.01 μM) for 24 h and DAPI staining was done and chromatin condensation was visualized by fluorescence microscopy. (TIF 565 kb) Additional file 2: Figure S2. Induction of apoptosis by DAT1 in colon cancer cell lines with wild type Ras and Raf. Left. Indicated cell lines were treated with 1.5 times IC_50_ concentration of DAT1 for a period of 24 h and stained with DAPI. Right. Apoptosis was quantitated by counting the number of cells with condensed chromosomes and percentage was calculated as compared to the total number of cells. Cells were counted from five different fields. Values expressed in the graph are the average values from two independent experiments. (TIF 902 kb) Additional file 3: Table S1. Acute toxicity studies of DAT1. Swiss Albino mice were divided into 6 groups of 6 mice each and were injected with either DAT1 or vehicle intraperitoneally. The mice were observed for a period of 14 days and the weights were taken before drug administration and after 14 days. Group I: 12 mg/kg, Group II: 20 mg/kg, Group III: 30 mg/kg, Group IV: 40 mg/kg, Group V: 50 mg/kg. (DOC 30 kb) Additional file 4: Figure S3. p53 activation in HCT116 cells. a. p53 activation was checked in HCT116 cells by western blot with phosho p53 antibody (Ser 15). b. Nuclear translocation of p53 was checked by immunofluorescence using an antibody against p53 and was visualized by Alexa 488. Nuclei were stained with propidium iodide. c. Nuclear localization was checked by western blotting against p53 antibody. An antibody against Lamin was used as a nuclear marker. (TIF 1599 kb)

## Abbreviations

ALP: alkaline phosphatase
ALT: alanine amino tranferase
AST: aspartate amino tranferase
BUN: blood urea nitrogen
DAPI: 4’,6-diamidino-2-phenylindole
DAT1: 4-Amino-5-benzoyl-2-(4-methoxyphenylamino) thiazole
DR4: death receptor 4
DR5: death receptor 5
ERK: extra cellular signal regulated stress kinase
JNK: c-Jun N-terminal kinase/stress activated protein kinases
MEK: mitogen/extracellular signal-regulated kinase
