# Supplementary figures and images for: ERK mediated upregulation of death receptor 5 overcomes the lack of p53 functionality in the diaminothiazole DAT1 induced apoptosis in colon cancer models: efficiency of DAT1 in Ras-Raf mutated cells
Source: Mol Cancer. 2016 Mar 8;15:22. doi: 10.1186/s12943-016-0505-7 (PMC4782294; doi:10.1186/s12943-016-0505-7)

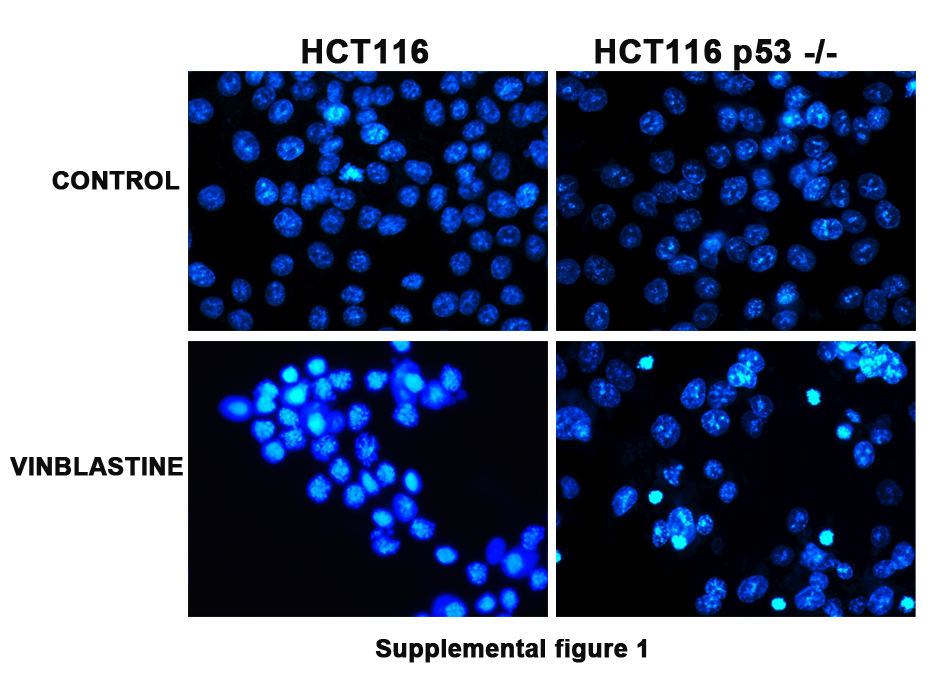

Supplement: Additional file 1: Figure S1. — Comparison of apoptosis in HCT116 and HCT116 p53 −/− cells treated with vinblastine : Cells were treated with vinblastine (0.01 μM) for 24 h and DAPI staining was done and chromatin condensation was visualized by fluorescence microscopy. (TIF 565 kb) [file 12943_2016_505_MOESM1_ESM.tif]

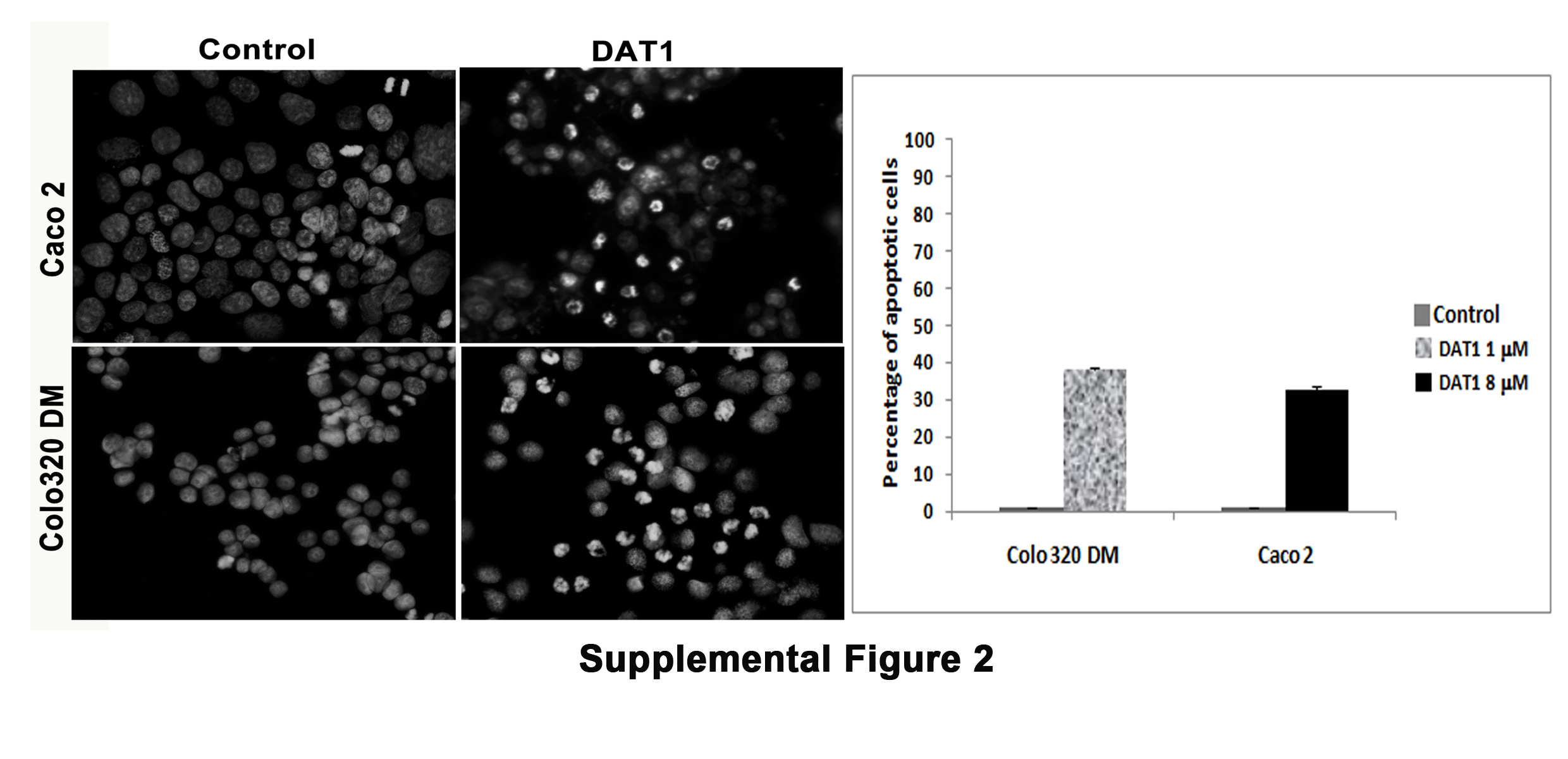

Supplement: Additional file 2: Figure S2. — Induction of apoptosis by DAT1 in colon cancer cell lines with wild type Ras and Raf. Left. Indicated cell lines were treated with 1.5 times IC50 concentration of DAT1 for a period of 24 h and stained with DAPI. Right. Apoptosis was quantitated by counting the number of cells with condensed chromosomes and percentage was calculated as compared to the total number of cells. Cells were counted from five different fields. Values expressed in the graph are the average values from two independent experiments. (TIF 902 kb) [file 12943_2016_505_MOESM2_ESM.tif]

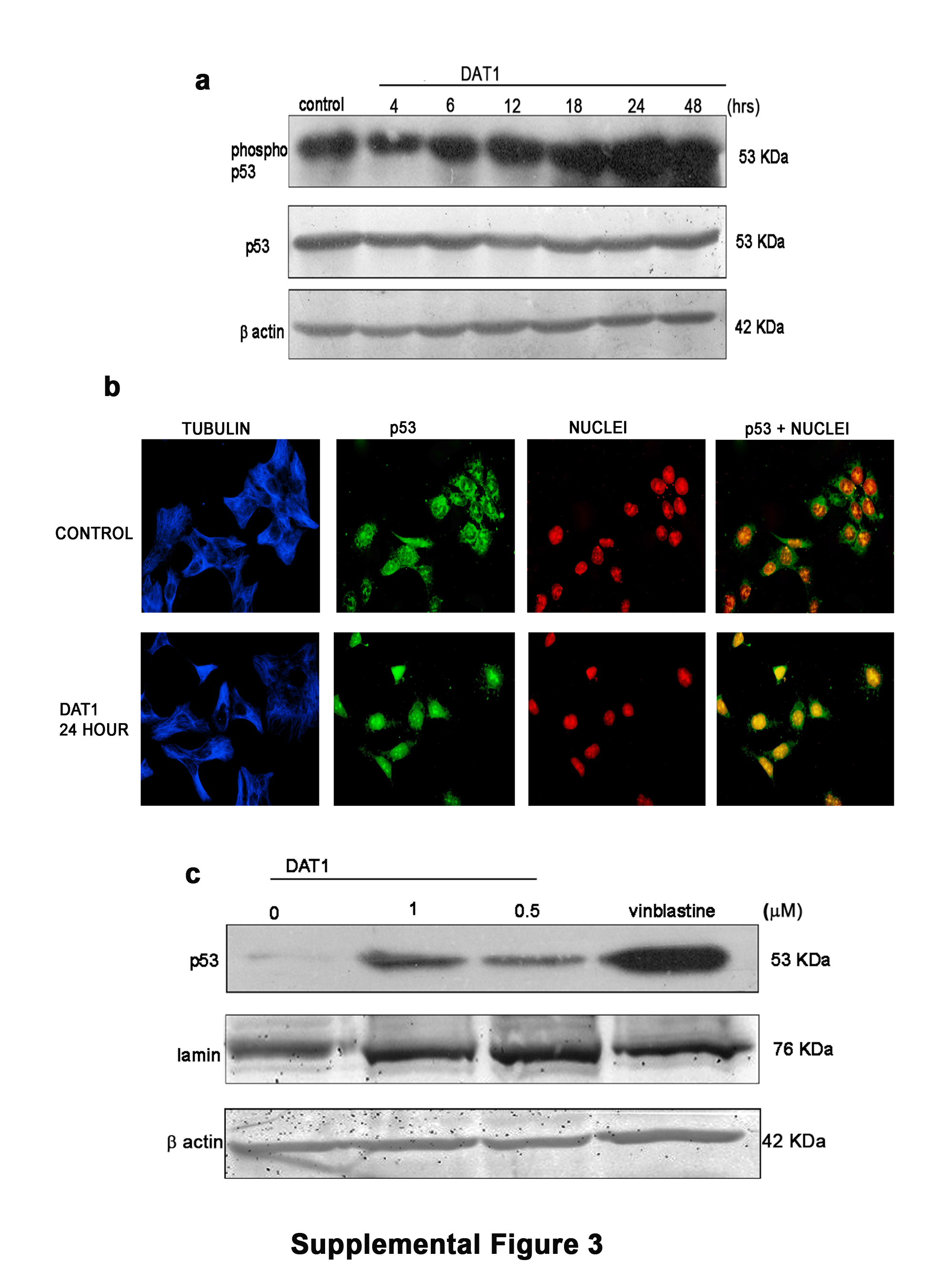

Supplement: Additional file 4: Figure S3. — p53 activation in HCT116 cells. a. p53 activation was checked in HCT116 cells by western blot with phosho p53 antibody (Ser 15). b. Nuclear translocation of p53 was checked by immunofluorescence using an antibody against p53 and was visualized by Alexa 488. Nuclei were stained with propidium iodide. c. Nuclear localization was checked by western blotting against p53 antibody. An antibody against Lamin was used as a nuclear marker. (TIF 1599 kb) [file 12943_2016_505_MOESM4_ESM.tif]
