# Supplementary material for: ERK mediated upregulation of death receptor 5 overcomes the lack of p53 functionality in the diaminothiazole DAT1 induced apoptosis in colon cancer models: efficiency of DAT1 in Ras-Raf mutated cells
Source: Mol Cancer. 2016 Mar 8;15:22. doi: 10.1186/s12943-016-0505-7 (PMC4782294; doi:10.1186/s12943-016-0505-7)
Supplement: Additional file 3: Table S1. — Acute toxicity studies of DAT1. Swiss Albino mice were divided into 6 groups of 6 mice each and were injected with either DAT1 or vehicle intraperitoneally. The mice were observed for a period of 14 days and the weights were taken before drug administration and after 14 days. Group I: 12 mg/kg, Group II: 20 mg/kg, Group III: 30 mg/kg, Group IV: 40 mg/kg, Group V: 50 mg/kg. (DOC 30 kb) [file 12943_2016_505_MOESM3_ESM.doc]

**Supplemental Table 1: Acute toxicity studies of DAT1**

Swiss Albino mice were divided into 6 groups of 6 mice each and were injected with either DAT1 or vehicle intraperitoneally. The mice were observed for a period of 14 days and the weights were taken before drug administration and after 14 days. Group I: 12mg/kg, Group II: 20 mg/kg, Group III: 30 mg/kg, Group IV: 40 mg/kg, Group V: 50 mg/kg

| **Groups** | **Initial weight**  **(g)** | **Final weight**  **(g)** |
| --- | --- | --- |
| Vehicle control | 28.2 ± 3.76 | 29.7 ± 3.78 |
| Group I | 32.4 ± 1.34 | 33.1 ± 1.56 |
| Group II | 32.8 ± 5.44 | 33.46 ± 3.87 |
| Group III | 27.2 ± 1.22 | 29.86 ± 2.82 |
| Group IV | 26.6 ± 2.4 | 27.8 ± 2.67 |
| Group V | 25.6 ± 2.03 | 26.12 ± 2.14 |
